# Evaluation of right ventricular pacing-induced coronary microvascular dysfunction using guide wire-based sensor technology: a case report

**DOI:** 10.1093/ehjcr/ytaf022

**Published:** 2025-01-22

**Authors:** Takashi Mishina, Naoya Inoue, Shuji Morikawa

**Affiliations:** Department of Cardiology, Chutoen General Medical Center, 1-1 Shobugaike, Kakegawa, 436-8555 Shizuoka, Japan; Department of Cardiology, Nagoya University Graduate School of Medicine, 65 Tsurumai-cho, Showa-ku, Nagoya, Aichi, Japan; Department of Cardiology, Chutoen General Medical Center, 1-1 Shobugaike, Kakegawa, 436-8555 Shizuoka, Japan; Department of Cardiology, Nagoya University Graduate School of Medicine, 65 Tsurumai-cho, Showa-ku, Nagoya, Aichi, Japan; Department of Cardiology, Chutoen General Medical Center, 1-1 Shobugaike, Kakegawa, 436-8555 Shizuoka, Japan; Department of Cardiology, Nagoya University Graduate School of Medicine, 65 Tsurumai-cho, Showa-ku, Nagoya, Aichi, Japan

**Keywords:** Right ventricular pacing, Chest pain, Coronary microvascular dysfunction, Ischaemic non-obstructive coronary artery disease, Case report

## Abstract

**Background:**

The diagnosis of ischaemic non-obstructive coronary artery disease is crucial for the differential diagnosis of chest pain. However, the pathophysiology of chest pain and evaluation of coronary microcirculation in patients with right ventricular pacing (RVP) have not been sufficiently reported.

**Case summary:**

The patient was a 53-year-old woman who underwent dual-chamber pacemaker implantation because of sinus node dysfunction. She experienced chest pain before pacemaker implantation; however, the frequency and severity of her chest pain increased after the implantation. She was referred for the evaluation of coronary microvascular dysfunction (CMD). Coronary angiography revealed no significant stenosis of the epicardial vessels. Subsequent evaluation of CMD showed that while the index of microcirculatory resistance [normalized index of microcirculatory resistance (IMR)] in the left anterior descending artery (LAD) was 19 U during the native rhythm, an increase in IMR (normalized IMR: 27 U) was observed during RVP.

**Discussion:**

Right ventricular pacing may not only induce left ventricular dyssynchrony due to non-physiological excitation propagation but may also provoke CMD in the LAD territory, particularly in the septal branches, which could contribute to pacing-induced structural CMD and chest pain. However, RVP may well be a contributing but not exclusively a factor of CMD.

**Learning points**

- Right ventricular pacing-induced dyssynchrony may trigger circulatory dysfunction in the septal region, potentially resulting in structural coronary microvascular dysfunction (CMD).
- Atrial fibrillation and increased heart rate may also contribute to CMD.
- Coronary microvascular dysfunction evaluation should be considered in patients with chest pain, wide QRS, left bundle branch block, or left ventricular dyssynchrony.

## Introduction

Ischaemia with non-obstructive coronary arteries has been widely recognized as a cause of chest pain, emphasizing the importance of evaluating coronary microcirculation in patients with chest pain.^1^ It includes categories such as epicardial coronary artery spasm, microvascular spasm, and coronary microvascular dysfunction (CMD). Hypertension, dyslipidaemia, diabetes, and menopause are common risk factors for CMD.^2^ Moreover, we previously reported that painful left bundle branch block (LBBB) syndrome is a unique condition that induces CMD.^3^

Right ventricular pacing (RVP), which has traditionally been used for bradyarrhythmias, results in an LBBB pattern with a wide QRS in ∼50% of cases, depending on ventricular lead placement; it has been reported to cause left ventricular dyssynchrony.^4^ Therefore, we hypothesized that a similar pathophysiology might apply to painful LBBB syndrome and RVP-induced LBBB in terms of their effects on coronary microcirculation. However, to the best of our knowledge, there have been no reports evaluating CMD using guidewire-based sensor technology in patients with RVP.

## Summary figure

**Figure:**
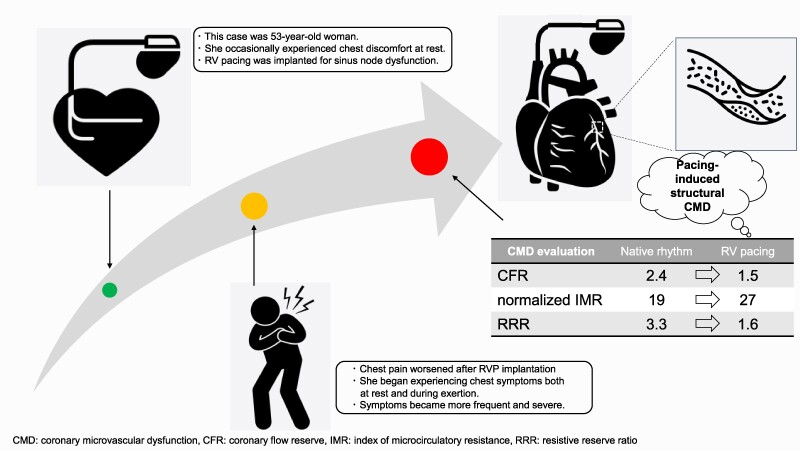


## Case presentation

**Figure 1:**
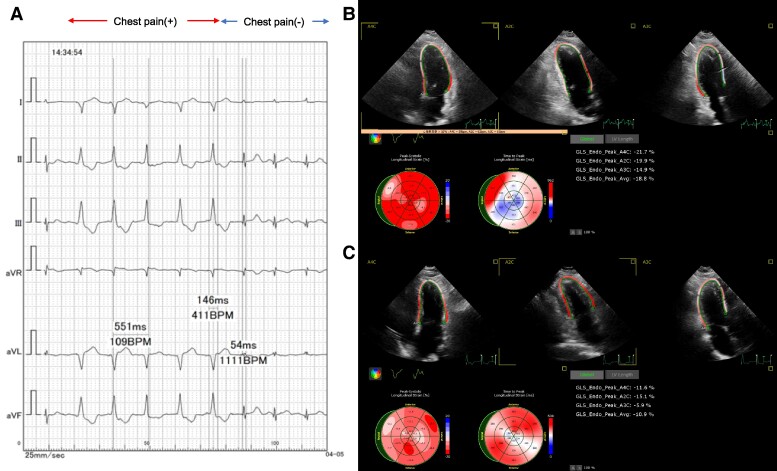
(*A*) ECG and presence or absence of chest pain during right ventricular pacing and native rhythm. (*B*) Strain analysis during native rhythm. (*C*) Strain analysis during right ventricular pacing (VVI—110 b.p.m.).

The patient was a 53-year-old woman with no history of hypertension or left ventricular hypertrophy. She underwent implantation of a DDD-RV pacemaker (Medtronic, Azure XT DR W2DR01) for the management of sinus node dysfunction (impedance at implantation: 560 Ω; RVP rate post-implantation: 15%). The QRS duration during the RVP was 146 ms (*Figure 1A*). On echocardiography, no left ventricular dyssynchrony was observed during native rhythm (QRSd: 54 ms), and the global longitudinal strain (GLS) was −18.8 (*Figure 1B*). However, abnormalities in left ventricular wall motion, such as apical rocking and septal flash (both indicative of dyssynchrony) appeared during RVP, while the GLS decreased, particularly in the septal region (GLS: −10.9; *Figure 1C*).

During follow-up conducted two months later, the patient reported chest pain, stating that she had experienced mild resting chest pain even before pacemaker implantation. However, the frequency and severity of the pain worsened after pacemaker implantation, regardless of activity or rest.

Therefore, we decided to perform coronary angiography, an acetylcholine provocation test, and CMD evaluation using guidewire-based sensor technology (CoroFlow, Abbott).

**Figure 2:**
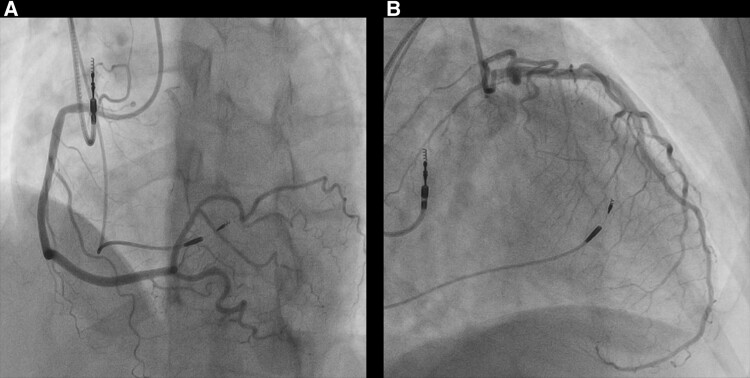
(*A*) Right coronary artery (LAO-CRANIAL view). (*B*) Left coronary artery (RAO-CAUDAL view).

Coronary angiography revealed no right coronary artery (RCA) stenosis (*Figure 2A*). Mild stenosis was observed in the left anterior descending artery (LAD) at Segment #7 (*Figure 2B*); however, the fractional flow reserve measured with nicorandil was 0.86. Subsequently, an acetylcholine provocation test (doses: RCA 20 and 50 µg, LAD 50 and 100 µg) was performed, but no electrocardiogram changes or coronary spasms of more than 90% were induced under angiographic guidance. However, atrial fibrillation (AF) was induced by acetylcholine administration (heart rate variability: 70–90 b.p.m.), and due to the inability to revert to sinus rhythm, evaluation of the coronary microcirculation was performed under AF after waiting for 10 min following the administration of nitroglycerine.

**Figure 3:**
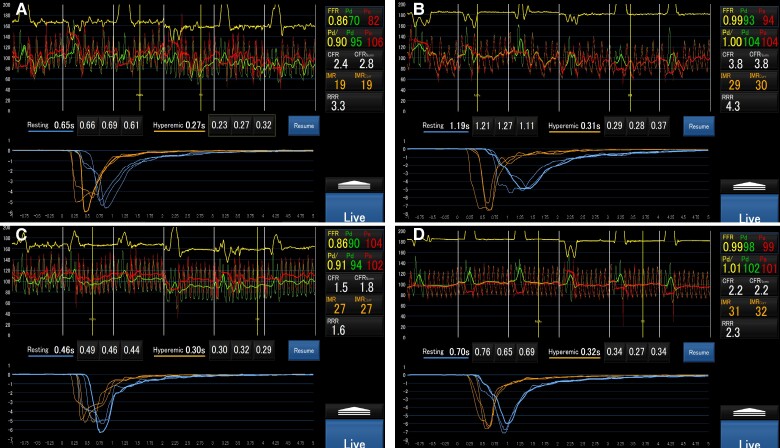
(*A*) CoroFlow evaluation of the LAD during native rhythm. (*B*) The RCA during native rhythm. (*C*) The LAD during right ventricular pacing (VVI—110 b.p.m.). (*D*) The RCA during right ventricular pacing (VVI—110 b.p.m.).

First, during native rhythm, a guidewire was inserted into the LAD for measurement, resulting in a coronary flow reserve (CFR) of 2.4, the index of microcirculatory resistance (normalized IMR) of 19 U, and a resistive reserve ratio (RRR) of 3.3 (*Figure 3A*). In the RCA under native rhythm, the results were CFR 3.8, normalized IMR 30 U, and RRR 4.3 (*Figure 3B*). To evaluate the impact of RVP on coronary microcirculation, the pacemaker mode was switched to VVI—110 b.p.m., and additional measurements were obtained for both the LAD and RCA. In the LAD, the CFR decreased, and the IMR increased, meeting the relevant diagnostic criteria (CFR < 2.0, IMR ≧ 2.5) for CMD (CFR, 1.5; normalized IMR, 27 U; RRR, 1.6; *Figure 3C*). In the RCA, the CFR was 2.2, the normalized IMR was 32 U, and the RRR was 2.3 (*Figure 3D*). In addition, chest pain was observed during RVP (*Figure 1A*).

## Discussion

This is the first report to evaluate the coronary microcirculation in two coronary arteries of a patient with RVP using guidewire-based sensor technology, demonstrating that RVP-induced electrical and structural effects can lead to CMD. Evaluating and diagnosing CMD is crucial because it is associated with an increased risk of major adverse cardiovascular events in patients who are positive for CMD.^5^

First, higher IMR values have been reported in the RCA compared with the LAD.^6^ Potential reasons include the spatial heterogeneity of myocardial blood flow in different vascular beds, which may elevate the IMR in the RCA, and differences in metabolism between the left and right ventricles, which may create disparities in myocardial blood flow between the LAD and RCA. Although the differences in results between these two arteries impact CMD diagnosis, recent studies have highlighted the importance of additional RCA measurements when CMD is negative in the LAD.^7^ Based on the RCA measurement results during the native rhythm in this case, CMD involvement cannot be disregarded as a contributing factor to chest pain before pacemaker implantation.

Subsequently, we considered the worsening of chest pain after pacemaker implantation and changes in LAD measurements during RVP. During RVP, as the right bundle is excited, delayed contraction of the left ventricular lateral wall occurs, causing the intraventricular pressure to shift towards the septum. These electrical and structural changes may impede coronary blood flow in the septal region.^8^ In this case, the worsening of GLS and the abnormal septal wall motion observed during pacing support this hypothesis. Ultimately, physical compression is thought to increase the IMR in the LAD, which is the main feeder of the septal branches.

A decrease in the CFR was consistently observed in both the LAD and RCA. A decrease in CFR is associated with one or a combination of the following conditions: (i) significant stenosis (>50%) restricting blood flow in the epicardial coronary arteries during maximum hyperaemia, (ii) increased resting blood flow, and (iii) suppressed increase in coronary blood flow during maximum hyperaemia. A limitation of this case is the concern about the impact of acetylcholine-induced AF and its high rate (VVI—110 b.p.m.) on the test results. Under such conditions, resting coronary blood flow may be unstable, and the reliability of the measurements can be compromised. Pintea Bentea *et al*.^9^ reported a consistent CFR decrease (18/18 vessels, 100%) and a high prevalence of structural CMD (47%) in patients with AF. However, their study did not compare with a sinus rhythm group, leaving unclear the precise mechanisms behind CFR reduction during AF or at high rates. Further validation is needed under controlled heart rate conditions, such as by incorporating tests at lower pacing rates (e.g. 80 b.p.m.).

Furthermore, in the present case, the screw-in lead was positioned near the RVOT. Although no cardiac tamponade was observed, and the impedance at implantation was within an optimal range, the possibility that the lead could have caused chest pain if screwed too deeply remained. Re-evaluation using an LAO 40° view would have been appropriate to verify these findings. Unfortunately, fluoroscopic images were not available, limiting our ability to confirm whether the lead was positioned on the free wall.

The pathophysiology of this case is complex, with AF and increased heart rate likely contributing to CMD. Additionally, pacing-induced dyssynchrony was observed, which, as previously reported by Yamano *et al*.,^10^ is also a likely contributing factor to CMD. These findings are consistent with our observations.

In conclusion, RVP may well be a contributing factor to CMD, but is not exclusive.

## Data Availability

The datasets are available from the corresponding author upon reasonable request.

## Lead author biography

Takashi Mishina is a cardiologist at Chutoen General Medical Center, Kakegawa, Shizuoka, Japan, where he worked from 2021 to 2024. He holds a medical degree from Showa University in 2019. He is currently focusing on the treatment of ischaemic heart disease and device therapy. In the future, he will be committed to being a pioneer in his practice, especially regarding INOCA and CMD.

## Notes

**Consent:** The authors confirm that written informed consent to submit and publish this case report, including the accompanying text and images, was obtained from the patient, as per COPE guidelines.

**Funding:** None declared.
